# Sport and Autism: What Do We Know so Far? A Review

**DOI:** 10.1186/s40798-024-00765-x

**Published:** 2024-10-03

**Authors:** Sidney Grosprêtre, Célia Ruffino, Cyrielle Derguy, Nicolas Gueugneau

**Affiliations:** 1 C3S, Culture Sport Health Society, University of Franche-Comté, Besançon, France; 2 Laboratoire de Psychopathologie Et Processus de Santé, Université Paris Cité, 92100 Boulogne-Billancourt, France; 3 ELLIADD, University of Franche-Comté, Besançon, France; 4 Institut Universitaire de France (IUF), Ministère français de lʼEnseignement supérieur, de la Recherche et de lʼInnovation, Paris, France

**Keywords:** Autism spectrum disorders, Asperger, Physical activity, Sports

## Abstract

Autism, or autism spectrum disorders, is a neurodevelopmental condition characterized by limitations in social interaction, communication skills, and repetitive behaviors. Although motor disorders were previously considered marginal in autism, recent research has highlighted their significance. Numerous studies have underscored the positive impact of sports on autistic individuals. This article presents a comprehensive overview of the literature regarding the effects of sport interventions on autistic individuals and aims to extract general and practical recommendations. Initially, the article reviews the various characteristics of autism that are positively impacted by sports, ranging from psycho-social skills to motor behavior. Subsequently, it examines how different configurations of sports practice (individual/collective, indoor/outdoor, etc.) may be suitable for autistic individuals. Literature research was conducted in two databases, resulting in the inclusion of 92 articles meeting longitudinal criteria (i.e., containing full sport/physical activity programs with pre-to-post analyses) out of 1665 initially identified articles. The findings suggest that individuals with autism can benefit from sports across a wide range of physical, psychological, and social factors. Importantly, there is no full contraindication for any activity, although some may require specific step-by-step preparation. Each activity has the potential to provide benefits in specific areas, as discussed in the article. In conclusion, further research is needed to explore the most effective strategies for implementing sports programs and maximizing their benefits for individuals across the autism spectrum.

## Key Points

- Autism, or autism spectrum disorders (ASD), was considered for years as a condition that only affected psychological factors and social skills, but recent evidence shows that a wider range of factors are affected, such as motor control.
- Sport is beneficial for autistic people, but there is a need to respect specific recommendations regarding dose and type of activity, that are still difficult to extract from the literature.
- Basically, there is no contraindication for any sport to be practiced with an autistic population, although each brings specific benefits and should need adaptations, which both need to be identified to optimize sports programs.

## Background

Sports science studies allow us to decipher the many determinants of performance, in order to optimize the training process and improve sensorimotor activity [1]. Educators or coaches can thus access a large body of evidence to help them develop athletes' behavior and match the requirements of a given activity. For instance, training load [2, 3], muscle contraction modalities [4, 5], or periodization [6, 7] are now well-defined features that contribute to the development of physical determinants. Also, cognitive factors such as attentional or mental processes [8, 9], or psychosocial features mediating the coach-trainee relationship and its environment [10–12], are characterized in many disciplines as they play a major role in performance.

However, this evidence-based approach remains to be widely developed when considering sport or physical activity in the context of neurodevelopmental disorders like autism. Indeed, while there is a broad consensus that autistic people could significantly benefit from sport or physical activity [13], a comprehensive model of intervention with precise recommendations is lacking.

Autism has an overall prevalence of 0.3–1.0% and covers a wide range of neuropsychological conditions impacting both individual and social functioning [14]. While the clinical manifestations are heterogeneous across individuals and age groups [15], autistic people consistently display qualitative differences in communication and social interaction, interests, sensory processing, and motor performance [16].

Social functioning in people with autism mainly reflects a lack of understanding others’ behavior, which can result from difficulties in effectively considering, interpreting, or reacting to the social and affective signals from others [17], for example, communication through eye contact or facial expressions. Reduced social skills can also lead to altered imitation performances, which negatively affect learning and interactions with caregivers and peers [18]. Verbal language might be absent, delayed, or specific in structure and content (e.g., presence of echolalia, disturbed prosody, restricted interest, and lack of reciprocity) [19]. In addition, autism may manifest deficits in sensorimotor control. For instance, limited motor coordination and deficiencies in fine and gross motor functioning [20, 21], or repetitive and stereotypical movements, were reported to be frequent (e.g., hand waving when excited or extraordinary postures in stressful situations [22]). Additional issues in maintaining balance and motion planning were also generally reported [23, 24].

Interestingly, several studies have reported motor deficits in relation to the severity of social and communication impairments [25, 26]. These results bring important arguments to consider sport and physical activity as effective intervention tools to address both social and sensorimotor skills in autistic people. Indeed, review articles have reported that various physical activity programs may significantly benefit sensorimotor function (e.g., muscle strength, motor coordination, aerobic fitness; [13, 27]), and also social functioning or communication skills in autistic populations (e.g., behavioral disorders, imitation skills, social awareness; [28, 29]). These data also suggest that physical activity or sport programs may have benefits for sensorimotor function and, therefore, may help to prevent chronic illness in a population that is often less active than non-autistic people [30]. In addition to improving social and communication skills [29], sport/physical activity may then contribute to enhancing the quality of life.

Nevertheless, despite an apparent consensus indicating that autistic people could benefit from physical activity-based intervention programs, there is still no work, to our knowledge, that could methodically guide the choices of medico-social actors, families, or sports educators for the practice of a specific sport in the case of autism. For instance, in specific chronic conditions, e.g., type 2 diabetes, cancer, or cardiovascular disease, patients and those around them generally know what and how to do in terms of physical activity because recommendations are clear [30]. To fill this gap, we attempted to provide a synthesis of the literature in a quantified manner, which generally aligns with sport science studies (frequency, duration, intensity of sessions).

In the first part of this review, the different factors of human performance (from psychological to physiological aspects) affected by autism will be explored. Then, in a second part, a review of the literature regarding longitudinal studies that evaluated the effect of a sport program on autistic individuals will be presented. In this part, sport will be compared with other types of intervention, using for instance the Applied Behavior Analysis (ABA). Finally, the last part will present practical recommendations extracted from this literature. Indeed, the main aim is to gather what recommendations can already be made from the existing literature to implement a sport program adapted to autistic people and, importantly, what remains to be investigated. Particularly, this last point is developed in the discussion section.

## Main text

### Methods

A literature search was conducted across two databases, PubMed and ScienceDirect, utilizing keywords such as 'physical activity,' 'sport,' and related terms ('sporting,' 'exercise,' etc.), linked with terms including 'autism,' 'autisms,' 'autistic disorder,' and 'autism spectrum disorders,' spanning from the earliest publications to April 2024. Initially, 1665 articles were identified (PubMed = 1,550 articles, ScienceDirect = 115). After removing duplicates, reviews, survey-based articles, acute studies, protocol trials, and articles retracted by editors or unavailable, a final count of 92 articles met our inclusion criteria. Specifically, we focused on articles employing a longitudinal approach, i.e., those implementing complete sport/physical activity programs with pre-to-post analyses. An exhaustive list of these publications is provided in Table 1.

**Table 1:** Characteristics of the selected articles

Reference	Autistic population	n	Mean age (years old)	Type of sport/intervention	Dose: number of weeks/frequency of sessions (duration of sessions)	Outcomes	Remarks
Adin & Pancar, 2023 [97]	Children	15	10–12	Swimming exercise	6-weeks/3 per week	Pulmonary Function Tests (forced vital capacity measurement, vital capacity measurement, breath inspiratory flow and peak expiratory flow calculation	Swimming exercise is effective in improving respiratory muscle strength and respiratory functions in children with autism
Ansari et al., 2021 [90]	Children	56	10	Compare the effect of a land-based and a swimming-based exercise program: karate exercise, aquatic training and control groups	10 weeks/2 per week (60 min)	Static and dynamic balance tests	Both interventions had a significant effect on balance abilities greater improvement in balance performance in kata techniques group
Arabi et al., 2019 [42]	Children	60	6–12	3 types: visuomotor, motor (group based), and computer-based training programs	10 weeks/3 per weeks (50 min)	- Visuomotor group decreased repetitive behaviors and increased gross motor skills - motor training group improved social behavior	
Arslan et al. 2020 [108]	Children	28	10	Circuit exercise program	12-week/3 sessions per week	Bruininks-Oseretsky test of gross motor proficiency (BOT-2) included running speed and agility, balance, bilateral coordination, and the standing long jump. Handgrip strength (both sides), reaction times (visual and auditory), and flexibility tests	The authors recommend that children with ASC start sports training immediately when diagnosed with autism and participate in structured physical activities with their peers
Bahrami et al., 2012 [41]	Children	30	5–16	Kata techniques program	14-week/56 sessions	Stereotypy	Teaching martial arts techniques to Children for a long period of time consistently decreased their stereotypic behaviors
Bahrami et al., 2016 [116]	Children and teenagers	30	5–16	Karate techniques training VS control	14 weeks/4 per week (90 min)	Reduction Communication deficit	
Barak et al., 2019 [82]	Adults	19	31	Game of Life (GOL) soccer	1 per week	Soccer skills, fitness and mobility improved	
Barrios-Fernández et al., 2022 [89]	Children and teenagers	52	6–12	Square-stepping exercise (SSE), a motor program initially created to strengthen the lower limbs of older adults	9 weeks/2 per week ( 30 min)	Balance, which will be measured with the Movement Assessment Battery for Children 2 (MABC2), and secondary outcomes will include sensory processing, attention, and executive functions	
Bass et al., 2009 [109]	Children	34		Therapeutic horseback riding	12 weeks	Greater sensory seeking, sensory sensitivity, social motivation, and less inattention, distractibility, and sedentary behaviors	
Brand et al., 2015 [70]	Children	10	10	Aerobic exercise training (AET) motor skill training (MST)	3 weeks/2 per week (60 min)	Sleep was assessed both objectively (sleep-encephalography [sleep-EEG]) and subjectively (parents' questionnaire). MSs were assessed via standardized test batteries. Parents completed sleep and mood logs, and ratings of mood	The pattern of results of this pilot study suggests that regular AET and MST impact positively on sleep, MSs, and mood among Children
Cai et al., 2020 [44]	Children	29	3–6	Scheduled mini-basketball training program	12 weeks/5 per week (40 min)	White matter integrity Social Communication	
Caputo et al. 2018 [48]	Children	26	8	Multisystem aquatic therapy	10 months/96 sessions	Behavioural, emotional, social and swimming skills	Aquatic therapy is useful for ameliorating functional impairments of children, going well beyond a swimming training
Carey et al., 2022 [64]	Children	24	11	Various PA program teacher-reporting group VS parent-reporting group	16 weeks/3 per week (60 min)	Anxiety Scale for Children for ASD (ASC-ASD)	Reduce anxiety Results demonstrate that 16 weeks, as opposed to 8, may be necessary to have a significant effect on in-school anxiety
Casey et al., 2015 [136]	Children	2	7 and 10	Highly structured therapeutic skating intervention	12 weeks/3 per week (60 min)	Pediatric Balance Scale, Timed Up and Go, floor to stand, Six-Minute Walk Test, goal attainment, and weekly on-ice testing	Improvements were found in balance, motor behavior, and functional capacity by posttest with gains remaining above pretest levels at follow-up Case study (n = 2)
Castano et al. 2023 [137]	Children	20	7	Structured physical exercise program	8-week/3 sessions per week (60 min)	Gross motor skills (Abbreviated Development Scale-3)	Improvement of gross motor skill after strctured physical exercise program
Clapham et al., 2020 [81]	Children	91	5–18	Surf therapy VS unstructured aquatic program	8 weeks/2 per week (60 min)	Pre and post physical fitness measures selected from the Brockport Physical Fitness Test	Significant improvements in core strength, upper body strength, cardiorespiratory endurance Significant reduction in total body fat %, more in the surf group Significant improvement in bone mineral density
Colebourn et al., 2017 [138]	Children	1	9	Gross motor intervention designed to improve the child's overhand throwing ability, which included weekly physical therapy instruction and daily throwing trials using applied behavior analysis approaches	20-week/1 per week (30 min)	Gains in throwing accuracy, significant gains on measures of the Bruininks-Oseretsky Test of Motor Proficiency-2, the Test of Gross Motor Development-2, and the School Function Assessment	
Columna et al., 2021 [91]	Children	15	4–11	Fundamental motor skills (FMS) intervention (home based VS workshop)	10 weeks/once per week	FMS using the third edition of the Test of Gross Motor Development	
Dehghani et al. 2023 [93]	Children	24	7–11	Multimodal exercise program (Sports, Play, and Active Recreation for Kids)	8-week/3 sessions per	Ground reaction forces and plantar pressure	Results suggest that a joyful and multimodal exercise program has positive effects on kinetic walking characteristics of autism spectrum disorder boys
Duan et al., 2022 [60]	Children	2	6	Adaptive rhythmic gymnastics (ARG)	12 weeks/3 per week (50 min)	ARG can boost the classroom participation of ASD children and improve their emotional problems Improve Joint attention	Case study on 2 children
Duffy et al., 2017 [31]	Children and teenagers	8	13–20	Therapeutic sporting programme	16 weeks/2 h per week	Gilliam Autism Rating Scale, 3rd edition was administered to identify and measure the severity of ASD behaviours at four time periods 6 subscales: emotional responses, cognitive style, and maladaptive speech	more severe cases of ASD did improve during the first 8 weeks
Edwards et al., 2017 [111]	Autistic and non-autistic children	30	6–10	Active video games (AVGs)	2 weeks/3 per week (45 min)	Actual (Test of Gross Motor Development) and perceived OC skills (Pictorial Scale of Perceived Movement Skill Competence for Young Children)	Actual skill scores were not improved in either group
Fragala-Pinkham et al. 2011 [83]	Children	11	7–15	Swimming and aquatic exercise	14 weeks/2 per week	Swimming skills, cardiorespiratory endurance, muscular endurance, mobility skills and participant and parent satisfaction	This pilot program was feasible and showed potential for improving swimming ability in Children
Garcia et al. 2024 [126]	Chidren and teenagers	18	13	Family jugo program	14 weeks	Sleep Quality	Participation in a family judo program may improve sleep quality in youth with ASD
Garcia et al., 2020 [43]	Children	14		Judo program	8 weeks/1 per week	Participants wore an Actigraph accelerometer to measure activity levels at baseline and post-judo	Percentage of time spent in daily MVPA (8% vs 4%, p = .05) increased following the intervention
Guan et al., 2022 [63]	Children	4	6	Roller-skating intervention, based on ABA prescription	8 weeks, 3 times/week (90 min)	Emotion regulation (sadness, anger, anxiety, fear). Negative emotion decreased after intervention	
Haghighi et al., 2022 [86]	Children	16	6–10	Combined physical training (CPT) (ball game, rhythmic movements, and resistance training)	8 weeks/3 per week	CPT program had a significant effect on indicators of social skills such as stereotypic behavior and communication, as well as PF such as handgrip strength, upper and lower body power, flexibility, balance, and agility	
Hassani et al., 2022 [92]	Children	30	8–11	Various physical activities	16 sessions (60 min)	Brininx-Oresetsky Test (BOT) and a program named "I can have physical literacy" (ICPL) and Sport, Play, and Active Recreation for Kids (SPARK)	
Hawks et al., 2020 [54]	Children and teenagers	15	7–16	iCan Bike Camp VS bicycle riding	1 week/5 per week (75 min)	Motor skill acquisition	Motor coordination and social communication correlated with rates of skill acquisition
Hayakawa & Kobayashi, 2011 [72]	Teenagers	23		Strenghtening Four special training machines were used for walking movements, for standing and walking balance, for leg-hip extension, and for ipsilateral movement in a sitting position	12 weeks/1 per week (30 min)	Significant improvement was observed in the 50-m dash, mean 10-m walk time, and 10-m obstacle course walk. The hip joint split angle showed a significant increase. Legal guardians all reported their child had "improved and/or progressed" for each of the targeted movements	
Helsel et al., 2023 [98]	Teenagers	20	11–17	Yoga	12 weeks/3 weeks	Anthropometric, motor skill, muscular strengh, static balance, dynamic balanc, flexibility, physical activity and sedentary time	These results demonstrate the feasibility and potential effectiveness of yoga to improve physical activity-related skills in adolescents with ASD
Hildebrandt et al., 2016 [139]	Young adults	43	23	Dance Movement Therapy	10 weeks/1 per week (60 min)	Scale for the Assessment of Negative Symptoms (SANS)	
Holm et al., 2014 [71]	Children	3	6–8	Therapeutic riding (horsback)	Multiple case design, with dosing of 1, 3, and 5 times/week	Parent-nominated target behaviors	Single subject multiple Baseline
Howells et al., 2022 [37]	Children	35	5–12	Football program	Various number of weeks (13 sesions on average)/max 1 per week (60–90 ùmin)	Caregiver-report using the child behavior checklist Vineland Adaptive Behavior scale increase in total MABC-2, aiming and catching, and balance scores for the intervention group,	
Ito et al., 2017 [140]	Teenagers	32		Dance exercise program	16 weeks/22 sessions	Calorie use during exercise and performance proficiency score	Significant positive impact on calorie use Not only autistic people
Jabouille et al., 2021 [88]	Children	2	10 and 7	Personalized balance rehabilitation program	4 weeks/2 per week	Those dual task (DT) conditions consisted of presenting images representing a neutral condition, sadness, anger, happiness, and fear. Postural control parameters (surface, velocity, medio-lateral and antero-posterior sway amplitudes of the center of pressure (CoP)	
Ji & Yang, 2021 [141]	Children	100	8–12	Physical football or virtual training	6 weeks/3 per week	Social Skills Improvement System Social-Emotional Learning Edition	Physical exercise and virtual training improve visual attention
Johnson et al., 2021 [125]	Children and their caregiver	10	5–11	Swimming program	12 weeks/3 to 5 per week (30 min)	Improved parental psychological health and child challenging behaviors	Future studies can focus on expanding the swim program to include all family members
Kawabe et al., 2022 [142]	Children	8	6–12	Esports program	12 weeks/1 per week (60 min)	Internet Gaming Disorder Test (IGD-20) scores, Kid-KINDL scores, and gaming time at home	
Ketcheson et al., 2022 [68]	Children	27	2 to 5	Mindfulness yoga program	12 weeks/1 per week (60 min)	Perceived stress, anxiety, and depression among urban caregivers of children on the autism spectrum decreased after inervention	Important to target parents Study only targeting parents’ perception
Ketcheson et al., 2022 [61]	Children	20	4–6	Motor intervention	8 weeks/20 h per week	Language skills (Mullen Scales of Early Learning) and motor skills (Test of Gross Motor Development-2)	
Koch et al., 2015 [143]	Young adults	31	22	Dance Movement Therapy	7 weeks/ 1 per week (60 min)	1. Heidelberger State Inventory (HSI)2. Questionnaire of Movement Therapy (FBT) 3. Emotional Empathy Scale (EES)4. Subscale social skills of the FBT	
Koehne et al., 2016 [144]	Adults	51	33	Dance movement therapy (DMT) VS. Control movement intervention	12 weeks/1 per week (90 min)	1. Multifaceted Empathy Test (MET) 2. Interpersonal Reactivity Index (IRI) 3. Assessment of Spontaneous Interaction in Movement (ASIM)	
Kozlowski et al., 2021 [80]	Children	58	7–12	Each session was manualized (operationalized instructional procedure and curriculum) and targeted components of fitness and motor performance using skill development exercises, workouts, and game-related activities	5 weeks/4 per week (60 min)	Fidelity (implementation accuracy), satisfaction surveys, attrition, and injuries Measures of work production (completed rounds of an exercise circuit) and within-session activity levels (time in moderate-to-vigorous PA), and six exercise tests (sit and reach, push-ups, sit-ups, air squats, long jump, and PACER)	
Lockard et al. 2023 [52]	Children to young adults	24	4–23	Judo	Not indicated	Social Skills Improvement System Social-Emotional Learning Edition	62.5% of parents agreed that their children demonstrated improvement across all six categories. The category with the greatest improvement was ‘Behavior at home’, while the category with the least improvement was ‘Eye contact’ Brief report with lack of information notably about the dose of practice
Lopez-Diaz et al., 2021 [46]	Children	13	6–10	Football training program	16 weeks/2 per week (60 min)	Generalized improvement in the dimensions linked to the social skills assessed	
Marouli et al., 2021 [145]	Children	8	6–14	Greek traditional dance	8 weeks/2 per week (40 min)	Educational Evaluation Tool for children with autism in the field of Social Skills	
Marzouki et al., 2022 [39]	Children	22	6	Two aquatic training regimens (technical vs. game-based) VS controls	8 weeks/2 per week (50 min)	Test of Gross Motor Development, the stereotypy subscale of the Gilliam Autism Rating Scale, and the Emotion Regulation Checklist	Improvement in gross motor skills was observed in both experimental groups compared to the control group Beneficial effects of aquatic activities on the motor and social skills that underpin 9the hypothesis that motor and intellectual domains are highly interrelated in autistic children
Mateos-Moreno et al., 2013 [146]	Young adults	16	25	Dance movement therapy	17 weeks/2 per week (60 min)	Evaluation of Autistic Behavior (ECA-R)	
Mastromicino et al., 2018 [147]	Young adults	56	22	Dance movement therapy	10 weeks/1 per week (60 min)	Cognitive and Emotional Empathy Questionnaire (CEEQ)2. Subscale Empathic concerns of the Interpersonal Reactivity Index (IRI)	
Miltenberger & Charlop, 2014 [47]	Children	3		Handball and 4-square		Athletic skills, speach	
Moradi et al., 2020 [148]	Children	100	6–9	Perceptual-motor exercises with or without hydroxycholecalciferol supplementation (D3 vitamin)	12 weeks/3 per week (60 min)	Stereotypical behavior	We concluded that combination of perceptual-motor exercises and vitamin D3 supplementation in Children leads to significant reduction in their stereotypic behaviors
Morales et al. 2022 [149]	Children	40	11	Adapted judo program	6 months	Gilliam Autism Rating Scale-Third Edition and the Test of Gross Motor Development	Improvement of score to the TGMD-3 and the GARS-3 three month after the end of the training program
Morales et al., 2021 [36]	Children	11	9–13	Judo intervention	8 weeks/1 per week (75 min)	Repetitive behaviours, social interaction, social communication, emotional responses, cognitive style and maladaptive speech scores	
Pan et al. 2017 [107]	Children	22	9	Physical activiy intervention involving table tennis	12 weeks	Motor skill (the Bruininks-Oseretsky OF Motor Proficiency, second edition) and executive function (Winsconsin Card Sorting test)	Physical activity interventions involving table tennis training may be a viable therapeutic option for treating children with autism spectrum disorder
Pan, 2010 [21]	Children	16		Water exercise swimming program (WESP) VS control	10 week	Improvement in aquatic skills and social behaviors	
Phung & Goldberg, 2019 [105]	Children	34	8–11	Mixed martial arts (MMA) intervention VS control	13 weeks/2 per week (45 min)	Executive functions (EFs)	Efficacious in meeting its goals of improving the executive functioning of Children
Pierantozzi et al. 2022 [150]	Children	21	11	Adapted jugo program	6 months/90 min per week	ALPHA-fitness battery; estimated VO2max and the waist/height ratio	A judo program tailored for Children can improve the cardio-metabolic health and cardiorespiratory fitness of its participants
Pitetti et al., 2007 [151]	Teenagers			Treadmill walking (TW) program VS control + Both groups continued to participate in their regular physical education curriculum	36 weeks	Exercise capacity and body mass index (BMI)	The TW program resulted in significant increases in mean monthly TW frequency, speed, elevation, and calories expended coupled with a reduction in BMI
Rafie et al., 2017 [123]	Teenagers	20	9–12	Motor activities, games and sports Selected physical exercise training	10 weeks/3 per weeks (45 min)	Childhood Autism Rating Scale (CARS) scores and level of abilities Bruininks-Oseretsky Test of Motor Proficiency (BOTMP)	Miscellaneous physical exercise programs which include ball games, delightful play and targeted play can improve perceptual-motor skills in adolescents with autism
Rafiei Milajerdi et al., 2021 [110]	Children	60	6–10	Two types of interventions, Sports, Play and Active Recreation for Kids (SPARK) and exergaming (Kinect) + control group	8 weeks	Motor skills (MS) and executive functions (EF)	This study suggests that structured physical activity (PA) interventions that target specific MS improve motor function in Children and exergaming could be effective for improving EF
Rivera et al., 2020 [43]	Children and teenagers	25	8–17	Judo program	8 weeks/1 per week	Parents of participants were given the Aberrant Behavior Checklist (ABC) to compare the severity of ASD-related behavior	No significant changes in ABC scores, however, parent interviews revealed that 78% of parents observed improvements in both social skills and self-esteem as a result of the judo program
Roșca et al., 2022 [87]	Children	28	8	Balance exercices	12 weeks/2 per week (30 min)	Decrease in surface of the confidence ellipse (A) and the length of the curve (L) described by the pressure center	Improvement in the balance of children with autism under complex evaluation conditions
Sansi et al., 2021 [56]	Non-autistic and autistic children	45	6–11	An inclusive physical activity (IPA) program	12 weeks/2 per week (60 min)	Motor and social skills	
Souza-Santos et al., 2018 [152]	Children	45	7	Dance program VS. Equine-Assisted Therapy VS. Dance & Equine- Assisted Therapy	12 weeks/2 per week (60 min)	Childhood Autism Rate Scale (CARS) 2.Functional Independence Measure (FIM) 3.World Health Organization Disability Assessment Scale RCT	
Sarabzadeh et al., 2019 [85]	Children	18	6–12	Tai Chi Chuan VS control	6 weeks/3 per week (60 min)	To assess motor skills, an M-ABC test	Significant difference in the subscales of ball skills and balance performance No significant difference in the manual agility scale
Shanok & Sotelo, 2022 [49]	Autistic individuals	15	-	Tennis-specific abilities	6 weeks/2 per week	Improvement in tennis skills Improvemet in hand–eye coordination (catching balls with one-hand) and leg strength (long-jump) Improvement in social skills, receptive communication skills	
Shanok et al., 2019 [55]	Children, teenagers and young adults	46	6–24	Golf-training program that integrates the teaching of autism-relevant social and communicative skills into each lesson	6 week/2 per week	Communication skills, social skills, motor skills, and regulatory skills improved	
Sotoodeh et al. 2017 [35]	Children	29	7–15	Yoga	8 weeks/24 sessions	Autism treatment evaluation checklist (ATEC)	This study provides support for the implementation of a yoga training program and identifies specific procedural enhancements to reduce the severity of symptoms in children with autism
Steiner & Kertesz, 2015 [120]	Children	26	Pupils	Therapeutic horse riding	4 weeks 1 per week (30 min)	Mental skills using Pedagogical Analysis and Curriculum (PAC) test consisting of four parts being communication, self care, motor skills and socialization. The Gait Cycle Analysis consists of the time-series analysis, the analysis of part of the gait cycle and the measurement of joint angles in each plane	Significant differences between before and after the therapy in the length of the gait cycle
Tabeshian et al., 2022 [34]	Children	21	9	Tai Chi Chuan training VS control	12 weeks/3 per week (45 min)	Stereotypic behavior measured using Gilliam Autism Rating Scale 2 Scores	Significantly altered by ~ 25% in the Tai Chi Chuan group
Thomas et al., 2016 [84]	Children	1	11	Behavioral skills training (BST) in skateboard		Percentage of correct skateboarding skills improved following BST	
Todd et al., 2010 [65]	Teenagers	3		Self-regulation instructional strategy on sustained cycling, which included self-monitoring, goal setting, and self-reinforcement	16 weeks	Development of self-efficacy	
Toscano et al. 2018 [79]	Children	64	6–12	PA program based on basic coordination and strengh exercise	48 weeks/2 per week	Metabolic profile, autism traits, and perceived quality of life	Physical activity, including basic coordination and strength exercises, as important therapeutic interventions for Children
Tse et al. 2019 [153]	Children	40	10	Basketball	12-week/2 sessions per week (total of 24 sessions)	Sleep paramaters (sleep efficiency, sleep onset latency, sleep duration and wake after sleep onset) and executive fonctions (inhibition control and working memory)	Improvement in sleep efficiency, sleep onset latency,sleep duration, inhibition control but not working memory
Tse et al. 2020 [154]	Children	27	8–12	Jogging	12-week/4 per week (30 min)	Emotional regulation checklist (ERC), CBCL	Significant improvement in emotion regulation and reduction in behavioral problems
Tse et al. 2022 [155]	Children	40	8–12	Running	12-week	Sleep and behavioral functioning	Findings of this study reconfirmed the sleep and behavioral benefits of exercise in children with autism spectrum disorder
Tse et al. 2022 [156]	Children	100	8–11	Jogging with or without melatonin supplement	10 weeks/5 per week	Sleep, endogenous melatonin level, physical activity level	The findings will provide evidence to determine whether physical exercise or melatonin supplement or the combination of interventions is the most effective to treat sleep disturbance in Children
Tse et al. 2023 [157]	Children	62	8–12	Cycling with or without melatonin supplement	10 sessions	Sleep	These findings suggest similar effectiveness of physical exercise and melatonin supplementation in improving sleep quality in Children
Tse et al., 2021 [106]	Children	62	9	Learning to ride a bicycle VS stationary cycling VS control	10 weeks/5 per week (60 min)	Four executive function components (planning, working memory, flexibility, and inhibition)	Significant improvements in all executive function components in the learning to ride a bicycle group (Ps < 0.05) but not in the other two groups
Wang et al., 2020 [40]	Children	33	3–6	Mini-basketball training program (MBTP) VS control	12 weeks/5 per week (40 min)	Executive functions and core symptoms (social communication impairment and repetitive behavior) by: Childhood Executive Functioning Inventory (CHEXI), the Social Responsiveness Scale-Second Edition (SRS-2), and the Repetitive Behavior Scale-Revised (RBS-R),	
Xu et al., 2019 [38]	Children	108	5	Sensory integration training and physical exercise intervention (A) VS only physical intervention (B)	12 weeks	Childhood Autism Rating Scale (CARS) and Autism Behavior Checklist (ABC)	Group A better than Group B
Yang et al. 2024 [158]	Children	30	3–6	Mini basketball program	12 weeks	The Social Responsiveness Scale-Second Edition (SRS-2) and Resting-state fMRI to evaluate ReHo	Effectiveness of a 12-week MBTP in ameliorating SCI and abnormalities in ReHo among preschool Children
Yang et al., 2021 [45]	Children	30	3–6	Mini-basketball training program (MBTP) VS control	12-week/5 per week (40 min)	Social Responsiveness Scale, Second Edition (SRS-2), whereas functional connectivity (FC) of the ECN was assessed using resting-state functional magnetic resonance imaging (rs-fMRI)	Enhanced functional connectivity between the right cerebellum and left inferior frontal gyrus
Yarımkaya et al., 2022 [159]	Families with autistic children	22	Families (child's age: 10)	Zoom-delivered physical activities VS control	10 weeks/4 per week (30 min)	Personal Information Form- Leisure Time Exercise Questionnaire-Semi-Structured Interview A significant increase was observed in the physical activity level of Children in the experimental group	
Ye et al., 2019 [96]	Teenagers	6	11–14	Aerobic, resistive, and neuromuscular exercises	8 weeks/2 to 3 per week (30–60 min)	Body composition and the Autism Treatment Evaluation Checklist (ATEC)	Fat mass of individuals with ASD were significantly reduced, and their behavior improved markedly, by all interventions
Yilmaz et al., 2004 [160]	Children	1	9	Swimming training	10 weeks/3 per week (60 min)	Swimming training is effective for development of physical fitness and water orientation	
Yu & Jee, 2020 [94]	Adults with mild and severe autism	35	20–29	Educational exercise program including ball games, skating, jumping	12 weeks/2 per week	Tests for physical fitness, including body composition and gross motor function (GMF) Mild ASD increased while Severe decreased or did not change	
Zanobini & Solari, 2019 [33]	Children	25	3–8	Swimming program vs control	Approximately 20 weeks/1 per two week (30 min)	Interpersonal skills, autistic mannerisms, and aquatic abilities motor skills	Positive changes in aquatic skills were maintained 6 months after the end of the program
Zhao & Chen, 2018 [57]	Children	41	6	24 exercise sessions targeting social interaction and communication of Children	12 weeks/2 per week (60 min)	Quantitative and qualitative instruments. SSIS and ABLLS-R results showed that an overall improvement in social skills and social interaction for the experimental group across interim and posttests	
Zhao et al., 2021 [53]	Children	61	6–12	Therapeutic horseback riding	16 weeks/2 per eek (60 min)	Overall social skills and communication, based on the SSIS and the ABLLS-R scores	
Zhao, You, et al., 2021 [69]	Parents of autistic children	75	Children: 6	Web-based parent–child physical activity program vs regular activities (control)	10 weeks/2 per week (60 min)	Mental health of parents of Children	

Our approach aims to address the relationship between previously implemented sport programs and autism by: (1) delineating the domains (social, motor, psychological, cognitive, etc.) impacted by sport participation in autistic populations; and (2) distinguishing between types of sports/physical activities (individual, collective, outdoor, indoor, etc.) and their respective effects. Additionally, we extracted general aspects such as training duration and the role of the coach in a final section.

### Modulation of Autism Characteristics Induced by Sports Practice

To introduce this section, the authors wish to inform the reader that they are aware that interventions aimed at modifying autism characteristics are subject to controversy. Indeed, there is increasing concern and advocacy from autistic communities, highlighting the potential harm of being forced to conform to allistic norms in social and communication behaviors. Therefore, in this section, reductions in composite autism scores are presented for indicative purposes only. We urge the reader to exercise caution in interpreting these results and to always prioritize the needs, desires, and interests of the individual with autism when proposing a sports activity, rather than focusing solely on the potential benefits observed in autistic manifestations.

#### Effects on Specific Characteristics

On the whole, engagement in sports activities has shown potential for optimizing various characteristics associated with autism [31]. These characteristics are commonly evaluated using validated questionnaires and scales, such as the Gilliam Autism Rating Scale, among the most frequently employed tools [32]. Typically, such scales comprise multiple items aimed at assessing and ultimately deriving a comprehensive score. However, scales like the Gilliam Autism Rating Scale often include subscales focusing on specific facets of autism, such as stereotyped behavior, communication, and social interaction. Numerous instances in the literature highlight how participation in sports can correlate with a reduction in the degree of autism characteristics, with examples ranging from swimming [33], tai chi [34], yoga [35], judo [36], or football [37].

Moreover, training targeted to specific skills (e.g., sensory or motor training) [39] or participation in generalized physical programs, lacking the structured aspect of a particular sport, have also shown great impact in modifying autism characteristics [38]. For example, aquatic training has been associated with a decrease in stereotypy, as indicated by reductions in the relevant subscale of the Gilliam Autism Rating Scale [39]. Stereotypy emerges as the characteristic most significantly impacted by sports participation across various disciplines [36, 39–41]. Notably, focused training in visuomotor exercises has demonstrated effectiveness in reducing stereotypic behaviors, whereas motor-only training yielded either minimal or no improvement [42]. These results suggest the complementarity between physical activity and more targeted interventions.

The potential impact of sports participation on autism characteristics is remarkable, with reported reductions in composite autism scores of up to 25% after three months of regular practice [34]. However, while behavioral changes are observed in some cases following training programs [43], this outcome is not universal. Factors such as the nature of the training regimen and, crucially, the dose–response relationship (i.e., the total duration in weeks and the frequency of sessions per week) are key determinants influencing the effectiveness of interventions. Adequate time investment appears necessary to produce significant changes for autistic individuals.

#### Social Aspects

Autistic individuals often contend with deficits across various social domains, primarily affecting communication and interpersonal interaction. A diverse array of sport activities has demonstrated efficacy in enhancing social and communication skills. These include team sports such as basketball [40, 44, 45], football [37, 46] and handball [47] or also more individual sports such as swimming [48], tennis [49], dance [50], martial art [51], judo [36, 43, 52], horseback riding [53], bike [54], golf [55]. Additionally, more generalized physical exercise programs [21, 56, 57] and various social sports games [58] have shown promise in fostering social skills. Notably, activities involving animal interaction, such as horseback riding [54, 60], have been particularly effective in promoting social engagement. Family-based sports games have also been observed to enhance communication skills in autistic children [59].

Moreover, the benefits of regular sports participation on social skills may extend beyond the sports arena. For instance, although studied on a limited scale, a rhythmic gymnastics program was shown to improve classroom engagement and attention in research conducted by Duan et al. [60]. The significant impact of sports programs on both motor and social skills underscores the intrinsic connection between these domains in autistic populations [39]. Lastly, even interventions solely focused on motor skills, devoid of structured gameplay or specific sports, have demonstrated potential in enhancing social interactions and language abilities [61].

#### Psychological Factors

Autistic individuals often grapple with challenges in regulating emotions, which can manifest in inappropriate responses. Additionally, autism is frequently associated with anxiety, stemming from feelings of social isolation, which can, in turn, elevate the risk of depression in some cases [19]. In addressing these concerns, engaging in sports activities can yield significant benefits, as physical exercise is well-established for its potential in alleviating symptoms of depression [62].

For example, interventions such as roller-skating [63], aquatic activities [39] or judo [36, 52] have been shown to improve emotion regulation. Moreover, participation in a diverse range of activities within a structured sports program can also help alleviate anxiety among autistic children [64]. However, Carey et al. suggest that to observe such effects, a certain duration of the program is essential, with noticeable improvements typically observed after 16 weeks rather than 8 weeks [64].

Interestingly, sports participation can also influence other psychological factors. Short-term training programs, for instance, have been linked to improvements in self-esteem [40]. Furthermore, self-efficacy, defined as an individual's perception of their own competence, can be enhanced through training programs that allow for self-regulated intensity [65].

#### Impact on Family and Caregivers

Autistic individuals' families, including parents, siblings, and caregivers such as teachers and professionals, are profoundly affected by their condition (e.g. [66, 67]). They often experience social isolation and psychological challenges such as anxiety, stress, and an increased risk of depression. Understanding the potential influence of sports activities on the well-being of autistic individuals' families and caregivers is therefore crucial. While research in this area is less extensive compared to studies on the effects of sports and physical activity on autistic individuals themselves, there are notable findings in the literature.

For instance, a study demonstrated that twelve weeks of yoga training resulted in reduced stress, anxiety, and symptoms of depression among urban caregivers of children on the autism spectrum [68]. This underscores the significance of considering the impact of sports programs not only on autistic individuals but also on their caregivers' perception of their children's progress and their own mental well-being. Importantly, it has been observed that parents' mental health may also benefit from engaging in sports activities with their autistic children [69].

Furthermore, parents' perceptions of their children's quality of life, mood [70], and autism-related behaviors [71] typically improve following their children's participation in sports programs. This trend extends to physical training programs, including strength and motor training, that may not necessarily incorporate a playful component [72].

#### Motor/Physical Performances

While representing a minority of the literature, it is now acknowledged that autism can encompass specificities in the physical and motor domains. Recent research indicates that 87% of individuals with autism exhibit motor impairments [73]. These impairments predominantly manifest in challenges related to postural control [74], gait [75], manual dexterity [76] or motor learning [77]. Moreover, autism is associated with challenges in strength, both in the upper and lower limbs, as well as in overall physical fitness [78]. This underscores the critical importance of implementing sports programs tailored to autistic individuals, with a focus on assessing these physical aspects during implementation.

Furthermore, individuals with autism tend to demonstrate notable improvements in strength and fitness adaptations in response to physical activity and sports. Engaging in various activities integrated into sports programs can enhance multiple facets of physical fitness in autistic individuals, including cardiovascular fitness [79], as well as strength in the upper and lower limbs [80]. Participation in a single sport can also enhance overall physical fitness, particularly if the activity encompasses various aspects of physical conditioning, such as surfing [81] or football [82].

Training in specific domains has been shown to lead to improvements in the trained tasks, as evidenced by enhanced aquatic skills following swimming programs [33, 83], soccer skills after soccer training [82], skating skills after a skating program [84], or tennis skills after a tennis program [49]. Moreover, studies indicate that gains in specific motor performances can translate to improvements in broader motor abilities, such as hand–eye coordination and lower limb power, as demonstrated by increased jumping abilities [49]. Activities requiring high levels of motor skills, such as golf [55], tai chi chuan [85], or ball games practice like football [37, 86], can improve various aspects of physical performance such as strength, flexibility, agility, and balance.

Balance training has shown promising results in reducing the area of displacements of the center of pressure in autistic children, who are often prone to balance disorders [87]. Similarly, activities emphasizing balance, such as skating, have been effective in improving this aspect [88], even at a lower training duration [88]. While activities targeting lower limb abilities, such as stepping exercises, may also benefit balance abilities [89], the transfer of gains from specific performances to general balance abilities in autistic individuals may be limited for certain activities, with swimming-based programs yielding lesser improvements compared to land-based programs [90].

In summary, motor skills benefit from training comprising specific motor skills exercises [91], but can also improve after more comprehensive physical activity programs [56, 92, 93]. Autistic adults have demonstrated improvements in gross motor functions, physical fitness, and body composition following physical activity programs, regardless of the severity of autism characteristics [94]. Additionally, changes in body composition, including reductions in fat mass, have been observed in autistic children following relatively short training durations [81], such as after a mixed aerobic-neuromuscular exercises training program [95, 96] or a mixed coordination-strength program [79]. This is in addition to improvements in pulmonary function after swimming programs [97], and metabolic aspects following longer-duration dance training [98]. Importantly, engaging in sports also influences daily physical activity, as evidenced by increased activity levels (monitored though actimetry) observed even after short-duration sports participation [99].

#### Cognitive Performances

Autistic individuals also face cognitive challenges, such as sensory integration issues [100, 101], difficulties in contextual interpretation [102], and impairments in executive functions [103]. Executive functions encompass various mental processes related to concentration, focus, and appropriate responses to external stimuli [104]. They comprise at least three core elements: inhibition, working memory, and cognitive flexibility. Inhibition enables selective focus on processing an external stimulus while suppressing others. Working memory involves holding and manipulating information in mind, such as utilizing stored data to solve ongoing problems. Cognitive flexibility refers to the ability to adjust perspectives according to external demands and produce appropriate reactions.

The executive functions of autistic individuals may benefit from sports and physical activities in general, although some activities may be more advantageous than others in this regard. For example, collective sports like basketball, involving ball manipulation, cooperation, and rapid perception and decision-making processes, engage high cognitive processes. Hence, a twelve-week basketball program tailored to autistic children might enhance cognitive functions, particularly working memory [40]. Similarly, combat sports like MMA, requiring quick decision-making processes, could improve working memory, cognitive flexibility, and to a lesser extent, inhibition in autistic individuals [105].

Specific enhancements in executive functions have also been observed after training focused on learning to ride a bicycle [106]. Interestingly, such improvements were not found in a group trained on a stationary bike, indicating that the motor learning process was more critical than the cycling activity itself. Furthermore, motor skill acquisition after learning to ride a bike has been correlated with improvements in social communication [54]. Table tennis has also been shown to effectively improve executive functions [107]. However, activities lacking fine motor skills or motor learning can still enhance cognitive functions. For instance, a stepping fitness program was found to improve sensory processing, attention, and executive functions [89]. Reaction times and cognitive flexibility improved after a circuit exercise program [108], suggesting that even simple physical programs devoid of specific strategies or fine motor skills aspects can benefit cognitive function. Additionally, a twelve-week horseback riding intervention resulted in reduced inattention and distractibility [109].

Interestingly, the balance between physical performance gains and cognitive enhancement in the autistic population may vary depending on the approach. For instance, while conventional sports activities may primarily enhance the motor domain, exergaming approaches (i.e., combining physical activity with video games) may be more beneficial for improving executive cognitive functions, particularly in autistic individuals [110]. It is worth noting that besides cognitive enhancement, active video games may also improve motor skills in autistic children, even after a short training period of six sessions [111].

It is now recognized that autism, as a neurodevelopmental disorder, has a physiological basis and exhibits specificities in brain composition and functioning [101]. Using functional magnetic resonance imaging, several studies have demonstrated significant changes in the brains of autistic participants after engaging in sports programs. For instance, basketball training programs have been shown to increase functional connectivity in the brain [112] and white matter integrity [44], both associated with observable improvements in cognitive abilities and behavioral factors such as communication skills. This underscores the potential for sports training to induce significant physiological changes in the brain, particularly since autism is associated with disruptions in brain connectivity [113, 114].

### What Type of Sport is Most Suitable and for What?

When implementing a sports program tailored for autistic individuals, various variables come into play. Here, we emphasize the variables that are most closely tied to autism characteristics. It is important to note that these variables are interconnected.

#### Individual or Collective?

Research has revealed that while more children tend to prefer individual sports, parents often perceive greater benefits with group sports [115]. However, each approach has its own advantages and drawbacks. Contrary to common belief, engaging in individual activities can also enhance social and communication skills, as demonstrated in programs such as martial arts [60] and dance [50]. For instance, karate has been found to reduce communication deficits in autistic children [106, 116]. However, some individual activities may have limitations in certain areas, such as empathy, as highlighted in a review by Chen et al. on dance programs [50].

As anticipated, group-based training programs tend to have a more pronounced impact on social behavior compared to individual practices, such as those done at home, without compromising the motor gains from training [42]. Therefore, it appears that engaging in activities with others, whether in collective or individual sports, can yield overall benefits. Furthermore, it has been suggested to integrate social sport games, which involve cooperation strategies, with individual physical or sensory training to enhance the effectiveness of sports programs in improving social skills [117].

#### Which Kind of Exercise Environment?

The type of practice environment, although very important, is scarcely studied in the literature or considered. Indeed, some autistic individuals might be sensitive to certain sensory stimuli, such as loud noises or bright lights. In this regard, it is advisable to recommend indoor activities. For instance, aquatic activities have been suggested to offer a sensory-friendly environment. Swimming pools often have a calm and predictable atmosphere, and the buoyancy of the water can provide a sense of weightlessness and freedom of movement [39].

However, while less predictable and controllable, outdoor practices are likely to be recommended for autistic individuals. Previous research has shown great effects of outdoor education on the social interactions of children with developmental disabilities [118]. The rationale behind this particular effect is not only based on the environment itself but also on the nature of the outdoor activities that are possible [119]. Indeed, horse-riding [53, 71, 109, 120] or bike riding [54] seem to be great tools for improving many aspects in the autistic population, from core social interactions to motor or cognitive aspects. Outdoor activities offer an environment with fewer boundaries than indoor practice. The adventurous side of an outdoor program, in addition to being well tolerated by autistic individuals under specific care, has been shown to provide excitement that leads children to communicate more [119].

Finally, intermediate solutions may also be proposed for autistic individuals who do not tolerate the uncertainty of such experience. For instance, less adventurous but still outdoor, golf practice offers a very calm, relaxed atmosphere [55]. This is the perfect example of an outdoor environment that is far more predictable and calmer than a crowded gymnasium with a very loud echo.

#### Game-Based, Technical or Fitness Centered?

Game-based sports prioritize the game itself, focusing on elements like scoring and team cooperation, while technical training emphasizes motor skills and abilities. Fitness-centered activities aim to enhance muscular function and overall fitness through exercises like strength training. Research has shown that regardless of the type of training—whether game-based or technical—an aquatic regimen yields similar gains in motor skills [39]. However, technical training may have more cognitive benefits compared to fitness-centered training [121]. Interestingly, the type of training doesn't appear to significantly impact general quality of life aspects, such as sleep or mood in autistic children, as both aerobic and motor skill exercises have been found to positively affect these domains equally [70]. Overall, while game-centered activities are often chosen for their playful nature and may not be solely focused on physical performance, they are still effective in improving motor skills [92].

Another aspect that can lead to specific improvements in the autistic population, particularly in cognitive and psychomotor domains, is the distinction between "open" and "closed" practices. In closed activities (e.g., track and field, gymnastics), participants hone their skills within a stable environment designed for their sport, aiming to master specific skills. For example, golf offers this closed practice type, providing a controlled training environment that may reassure participants. Conversely, open practices (e.g., collective sports) involve high levels of uncertainty regarding the environment or the actions to be performed. Despite concerns about the feasibility of open practices for autistic participants due to their unpredictable nature, research has shown that many open practices are beneficial for this population across various domains, from social to motor skills. Numerous studies on team sports, exemplified by basketball [40, 44, 45], football [37, 46, 82, 122], or handball [47], support the effectiveness of open-skilled sports. Moreover, activities with greater uncertainty, such as ball games, may be particularly beneficial for perceptual-motor skills in autistic individuals [123]. This is also observed in individual activities characterized by high levels of uncertainty, such as boxing [105] or tennis [49].

#### Which Type of Partner?

When integrating autistic individuals into sports practice, various approaches can be taken in organizing the group dynamics within the lesson. Essentially, four forms are commonly observed: (1) groups comprised solely of autistic participants, (2) mixed groups of autistic and non-autistic individuals, (3) one-to-one interactions involving a coach and an autistic participant (private lessons), or (4) activities that involve family members (parents, caregivers, siblings) in the practice. However, there is limited research comparing the effects of sport programs across these different modalities.

Community sport programs, which involve a group of autistic participants, have demonstrated positive effects on autism characteristics such as social and behavioral functioning and communication [35]. While one-to-one programs (involving one coach and one participant), or even home-based training, have also shown positive effects, they often require additional interventions aimed at developing social skills. Collective activities inherently promote social interaction, although for individuals with lower-functioning profiles, it may be necessary to initially focus on individual practice. Involving parents and/or siblings can be an effective way to foster the interest of autistic individuals in sports activities [59].

Numerous studies have assessed the effects of family-based programs, involving siblings and/or parents, all of which seem to agree on the particularly positive impact they have on various aspects of autism in the participants [124], 125. Family-based sports programs may yield benefits beyond social aspects, such as improving sleep quality in autistic children, as evidenced by shared swimming programs [126]. Additionally, it has been observed that parental mental health may benefit from engaging in sports activities with their autistic children [69]. Importantly, while mixing autistic and non-autistic individuals requires careful consideration and preparation, it is believed to have very positive effects, including fostering positive attitudes among non-autistic individuals toward their autistic peers [56].

Lastly, as mentioned earlier, animals can also serve as excellent training partners. Animal-assisted interventions have shown significant benefits across cognitive, psychological, and core characteristics of autism [53]. Specifically, horseback riding, besides its positive effects on cognitive, motor, and social factors, offers opportunities to develop relationships with horses and to learn about animal care and behavior.

### Sport Versus Conventional Interventions

Before discussing the potential implementation of sport programs on autistic people and their effects on many outcomes related to autism, we propose to compare the effects of sport programs to other conventional interventions. Recent meta-analyses thus allow us to put in perspective the effects of Applied Behavior Analysis (ABA) and medication for autism [127, 128] in regard to sport-based interventions [129]. These studies quantified the effects of a specific intervention on clinical symptoms compared with a control or placebo intervention in terms of a size-effect measure, i.e. standardized mean differences (SMD) with a 95% confidence interval (CI). ABA is a well-known behavioral method that consists of analyzing how the individual’s environment influences their behavior [130] and describes interventions applying the findings of such analyses to change behavior [131]. It is based on operant conditioning and aims to assess and change challenging behavior (e.g. toilet training) as well as to promote and generalize more adaptive behavior, for example, by using systematic reinforcement. Pharmacological studies are scarce but a few studies have evaluated the effect of different drugs (mainly antipsychotics) on restricted and repetitive behaviors; i.e. stereotypy [128].

The meta-analysis by Eckes et al. [131] revealed that ABA-based interventions improve adaptive behavior more strongly compared to the usual intervention, minimal intervention, or no intervention at all (nine studies with 547 participants, SMD = 0.37, 95% confidence interval (CI) [0.03; 0.70]). Adaptive behavior was mostly measured with the Vineland Adaptive Behavior Scale (VABS; [132]) and combines communication ability, social interaction ability, daily living, and motor skills. Intellectual functioning (verbal comprehension, reasoning, knowledge, and memory) also showed a significant improvement after ABA-based interventions as compared to control groups (eight studies with 293 participants, SMD = 0.51, 95% CI [0.09, 0.92]). Concerning language ability and autism characteristics there is no strong evidence for improvement in children receiving ABA-based treatments compared to children in other intervention conditions (respectively five studies with 210 participants, SMD = 0.30, 95% CI [− 0.13; 0.72] for language ability; and three studies with 107 participants SMD = − 0.26, 95% CI [− 0.60, 0.07] for autism severity).

Interestingly, these data could be put into perspective by the study of Huang et al. [129] which synthetized the effect of sport-based interventions on autism characteristics. They revealed significant improvements in adaptive behavior, specifically in social interaction (three studies with 197 participants, SMD = 0.58, 95% CI [0.29; 0.87]), and communication abilities (4 studies with 240 participants, SMD = 0.29, 95% CI [0.04; 0.55]). Nevertheless, they reported no strong evidence for a decrease in autism characteristics manifestation after sport-based programs (four studies with 172 participants, SMD = 0.17, 95% CI [− 1.46; 1.11]).

Concerning the restricted and repetitive behaviors of autistic population, the studies of Zhou et al. [132] and Huang et al. [129] allow us to compare the effect of medication and sport-based activities, respectively. Remarkably, antipsychotics significantly improved restricted and repetitive behaviors outcomes compared to placebo (64 studies with 3499 participants; SMD = 0.28, 95% CI [0.08; 0.49]), while sport-based programs have been shown to only have a moderate effect (12 studies with 146 participants; SMD = 0.13, 95% CI [− 0.46; 0.20]).

Despite the heterogeneity of experimental designs, these data make it possible to understand the orders of magnitude of the effects of different interventions on several autism characteristics. However, other meta-analyses are still needed with the specific objective of precisely comparing the effects of different interventions on autism outcomes, which is beyond the scope of our study.

## General Recommendations

Traditionally, the World Health Organization (WHO) recommends that individuals engage in at least 150 min of moderate-intensity or 75 min of vigorous-intensity physical activity per week. However, determining the ideal dose of physical activity for autistic individuals remains a challenge, as it may vary depending on factors such as age, abilities, and health status. Generally, autistic individuals tend to participate at lower levels compared to their peers [133]. As previously discussed, they face cognitive, emotional, communicative, social, and movement challenges that affect their daily functioning, learning, and leisure activities [89]. The duration of training can have varying effects on these aspects. Research suggests that longer durations, such as 16 weeks instead of 8, may be necessary to significantly impact in-school anxiety [64]. In the cognitive domain, changes may occur relatively quickly with specific training focused on this aspect. For example, active video games, which provide strong cognitive stimuli, may enhance cognitive skills after just 6 sessions [111]. However, motor skills may require a longer training duration to show improvement. Additionally, the frequency of sessions per week plays a significant role in the beneficial effects of a sport program. Studies have shown that increasing the frequency from 1 to 3 to 5 times per week significantly enhances the magnitude of observed changes [71].

In the literature reviewed, a wide range of training durations and organization strategies were found, without a consensus reached on optimal practices. The average duration of protocols was 12.2 ± 7.7 weeks, with a mean frequency of 2.6 ± 1.4 sessions per week. Session duration averaged 56.5 ± 19.3 min, often falling short of recommended guidelines when considering exercise intensity, which is not consistently reported or quantified.

This highlights the need to carefully design sport programs, considering factors such as duration, frequency, volume, and intensity. Flexibility is crucial given the population's specificity, necessitating adaptation to individual needs and abilities. This may involve modifying activities or providing accommodations, such as additional time or support. Listening to the individual's needs and preferences and being open to trying different approaches is essential.

Many autistic individuals benefit from structured and predictable environments, clear routines, and expectations. Providing schedules or step-by-step instructions can help them understand what to expect. Breaking down new skills into small, incremental steps and offering explicit instruction is important. Simple, incremental feedback using verbal, visual, and manual cues is effective. Positive reinforcement, such as rewards or encouragement, can motivate and support learning and progress.

Autistic individuals may experience anxiety, frustration, or other emotional challenges during sports or physical activities. Discussing their needs and goals with coaches or instructors beforehand and establishing clear communication channels can help address these challenges.

Considering sensory needs and preferences when selecting activities and providing a sensory-friendly environment, if necessary, is crucial. Activities should be enjoyable, suitable, and safe for the individual, with necessary safety equipment and supervision provided. Sensory issues should be addressed promptly as they arise.

## Discussion

This article aimed to provide an overview of the literature on the implementation of sport programs for autistic individuals and their effects on multiple aspects. Importantly, it seeks to extract established recommendations for these populations and identify areas that still require further research.

Across the literature reviewed, one can observe the diversity of sport practices examined. No specific type of activity stands out, indicating that a wide range of sports are suitable and well-tolerated by autistic individuals. Whether it is a collective or individual sport, involving confrontation or cooperation, practiced outdoors or indoors, the choice of a sport activity appears to primarily depend on the preferences of the individuals involved. Overall, sport participation has shown high levels of acceptance based on satisfactory surveys, including feedback from both autistic and non-autistic participants, as well as their relatives [80]. These findings support Chan et al.’s conclusion that *“given their affordability, versatility, and efficacy, physical activity interventions could be considered a cost-effective option for autism spectrum disorder management in the future”* [134].

The literature on sport and autism has grown exponentially in the last decade (from fewer than 20 articles published and referenced in PubMed in 2013 to 153 in 2023). However, there is still much information missing. It is noteworthy that many studies have small sample sizes, some with as few as 2 or 3 participants, indicating a need for larger-scale studies. Additionally, cultural differences may exist in the implementation of sport programs and consideration of autism, with variations from Asia (where many studies have been conducted) to Europe. The age range of the population studied in the literature is also wide, but the focus has predominantly been on children. Specifically, 81% of the literature screened tested autistic populations aged 3–12 years old, while only 7% focused on adults. Despite representing a larger proportion of the autistic population, there is also an overrepresentation of males in the scientific literature. Therefore, there is a need to address gaps in the literature regarding autistic populations. Furthermore, the definition of autism itself may vary between studies, with autistic individuals sometimes included in a broader range of other neurodevelopmental disorders, such as ADHD (attention-deficit/hyperactivity disorder). However, few studies have assessed or compared the effects of sport interventions on different levels of autism severity, as evaluated by scales like the Gilliam Autism Rating Scale or the Childhood Autism Rating Scale (CARS). Although severe forms of autism may require more attention and intermediate steps to achieve autonomous sport practice, such as starting with the presence of parents and/or siblings, it appears that individuals across all profiles of autism might benefit from physical activity and sport to a similar extent [94].

Across the whole literature on sport/physical activity and autism, the proportion of studies employing objective and quantitative markers to assess the effects of a given sport program on autistic participants remains low (less than 20 percent of the literature on sport and autism). The majority of the literature comprises cross-sectional analyses (group comparisons) or survey analyses, indicating a need for further objective evidence regarding the long-term effects of sport practice through the implementation of more longitudinal studies involving cohorts of participants [58]. The progression of outcomes should be compared to a control group or participants engaging in another sport program, a practice that is not consistently employed (often limited to case studies, single-group studies, small samples, etc.). While most studies focus on a single sport or type of intervention, it is recognized that autistic individuals may exhibit a variety of responses to sport, and what works for one individual may not work for another [135]. Studies incorporating different forms of practice have provided valuable insights, revealing specific gains according to the nature of the practice. While previous research generally suggests that engaging in any sport may lead to a general improvement across various outcomes, particularly in autism characteristics, specific effects have been observed. For example, studies comparing different interventions, such as technical versus game-based approaches [39], aerobic versus motor skills exercises [70], or different types of environments [90], have identified benefits across all forms of practice in psycho-social skills, with some specific effects observed in motor skills and neuromuscular aspects.

Another crucial aspect of the literature on sport and autism pertains to the types of outcomes evaluated. There exists a wide range of outcomes, including autism characteristics and social skills, psychophysiological factors, and neuromuscular components of human performance. However, the broad variety of interventions and populations tested makes it challenging to gain a clear understanding of the effects of sport practice on these numerous factors. Few studies are directly comparable, with the most commonly evaluated factors being autism characteristics themselves, such as social and communication skills, using validated questionnaires and scales. Psychological factors and the well-being of participants and relatives are also frequently assessed, and these factors are consistently influenced by sport practice. However, literature on the effects of sport practice on motor skills, cognitive factors, neuromuscular plasticity, or the physiology of autistic populations remains scarce. Yet, there is increasing evidence suggesting that these domains should be given as much consideration as psycho-social factors. Importantly, it should be noted that all factors are interrelated; for example, promoting muscle strength gains in autistic individuals may lead to gains in psychomotor functions [78].

Specifically, regarding motor skills, it is noteworthy that many studies assess them subjectively using evaluation scales. There is a need to standardize a more formal and quantitative evaluation by employing functional tests or analytical performance measurements (e.g., strength, speed, etc.). Taking a more fundamental perspective, incorporating objective measurements of cognitive, cardiovascular, and neuromuscular functions into the evaluation of the impacts of sport programs should contribute to a better understanding of the motor impairments observed in autistic populations. This is particularly important considering ongoing discussions about the need to include the motor domain in the definition of autism [61].

**Fig. 1:**
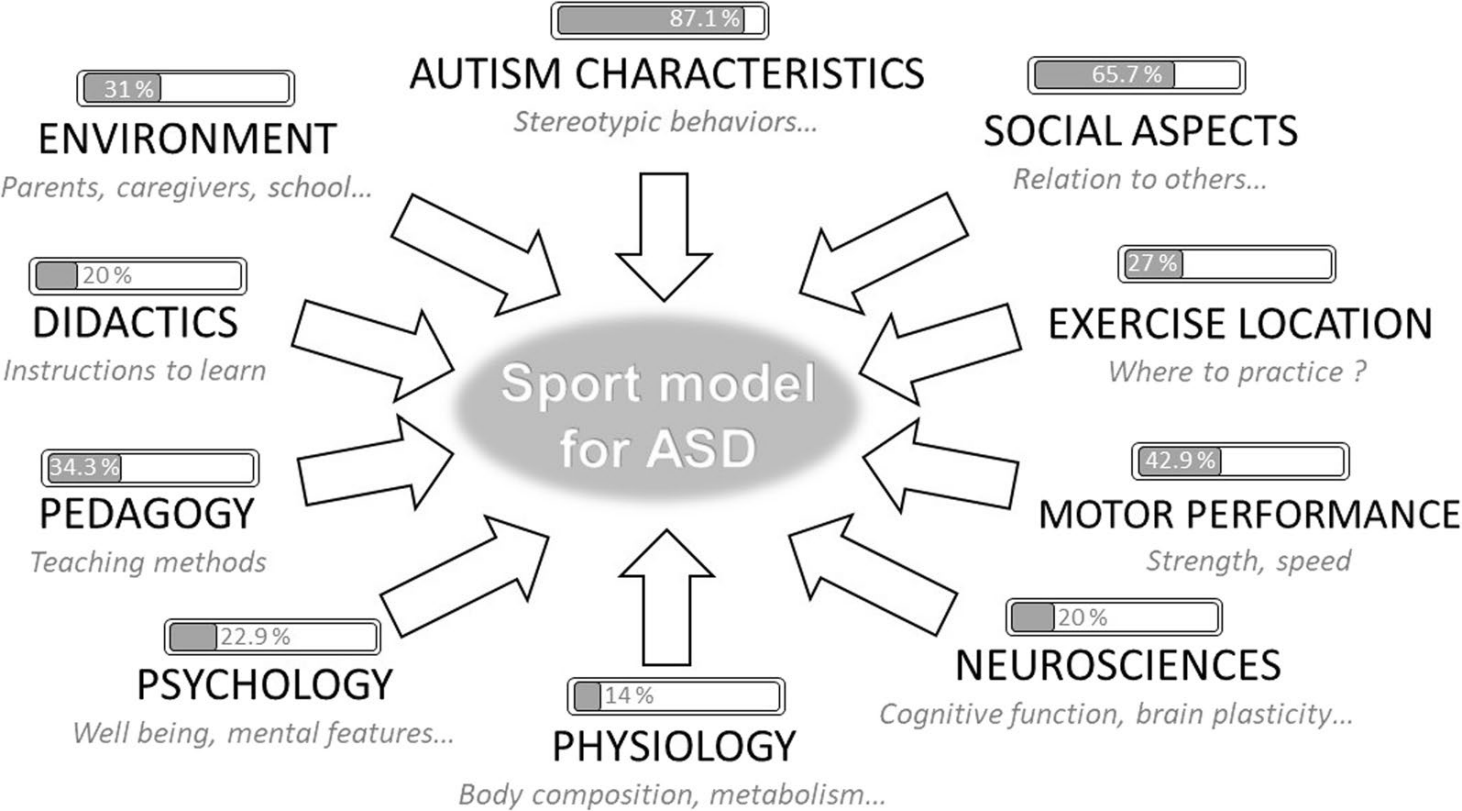
Overview of the proposed factors to consider when developing a sport model for autistic people. Each factor that should be considered when analyzing/implementing a sport program is indicated at the end of each arrow. Bars represent the percentage of articles of all articles screened that took the factor into consideration, as an outcome or as an independent variable. Then each bar represents the degree of consideration in the current literature, highlighting strengths and weaknesses of the actual knowledge

Finally, based on a holistic approach, Fig. 1 depicts factors that should be considered when constructing an adapted sport program designed for autistic individuals. All elements extracted from the literature that are deemed important to consider are integrated, along with information on the proportion of consideration in previous works to date.

## Conclusion

In conclusion, authors unanimously agree on two points: (1) sport and physical activity are fundamental components of the quality of life for autistic individuals and their families, and (2) there is still a significant gap in knowledge regarding this topic. The diverse array of studies, encompassing various sports, populations, and tested outcomes, makes it challenging to reach a consensus on many aspects. Moreover, there are crucial practical considerations that need scientific elucidation in constructing physical activity programs, such as determining the optimal training volume (session duration, frequency, etc.) and exercise intensity.

To address these gaps, it is imperative to bridge the scientific understanding of autism from a clinical perspective with the principles of exercise physiology. Studies examining sport and autism would benefit from incorporating objective markers of cognitive and motor performance, as well as tools to monitor training load and assess individual responses to physical exercise. This approach would facilitate the customization of sport programs, ensuring a relevant progression throughout the program to maintain motivation, enjoyment, and seek optimal benefits. Ultimately, the goal is to cultivate a long-term commitment to the benefits of sport practice across the lifespan, rather than merely focusing on the duration of a specific program.

## Data Availability

Not applicable.
